# A novel full digital workflow for dental autotransplantation: surgical accuracy and clinical outcomes in a prospective cohort study

**DOI:** 10.3389/froh.2026.1786907

**Published:** 2026-04-10

**Authors:** Marianna Salviati, Alessandro Antonelli, Vincenzo Cosentino, Luca Boschini, Michele Melillo, Selene Barone, Amerigo Giudice

**Affiliations:** 1 Department of Health Sciences, Magna Graecia University of Catanzaro, Catanzaro, Italy; 2 Department of Clinical and Experimental Medicine, University of Foggia, Foggia, Italy

**Keywords:** 3D-printed surgical guides, CBCT-guided planning, dental autotransplantation, donor tooth, full-digital workflow, periodontal ligament preservation, surgical accuracy

## Abstract

**Introduction:**

Dental autotransplantation is a biologically conservative alternative to prosthetic or implant-supported rehabilitation, though achieving predictable outcomes remains challenging. The use of digital workflows integrating CBCT-based planning, 3D modelling, and customized surgical guides may improve surgical precision and reproducibility. This study aimed to evaluate the accuracy and clinical outcomes of a full-digital workflow for dental autotransplantation.

**Methods:**

Patients with a non-restorable tooth and a healthy third molar suitable for donation were included. Preoperative CBCT and intraoral scans were used to enable automatic segmentation, virtual simulation of donor tooth positioning, and planning of alveolar site preparation. Customized, root-based surgical guides were then designed and 3D-printed. The surgical procedure included extraction of the non-restorable tooth, harvesting of the donor third molar, and its transplantation into the prepared alveolar site. The accuracy of the surgical protocol was assessed by superimposing postoperative CBCT-derived 3D models onto the preoperative plan using digital qualitative and quantitative analyses. Clinical outcomes, including tooth stability, periodontal health, and periapical status, were evaluated over an 18-month follow-up period.

**Results:**

Ten patients (6 males, 4 females; mean age 27.2 ± 2.77 years) were enrolled. Mean surface deviations between planned and post-operative models were 0.63 ± 0.45 mm (tooth) and 0.17 ± 0.06 mm (alveolar bone). Three-dimensional linear discrepancies averaged 2.04 ± 1.24 mm at the coronal level and 1.36 ± 1.00 mm apically. Angular deviations showed no systematic rotational bias, with limited pitch and roll deviations and higher interindividual variability for yaw. Two teeth required post-surgery endodontic treatment. All transplanted teeth showed clinical stability, physiological probing depths, and absence of pathological mobility.

**Conclusions:**

At 18 months, a fully digital workflow with customized surgical guides enabled accurate and minimally invasive dental autotransplantation, reducing extra-alveolar time and preserving the vitality of the periodontal ligament.

## 1 Introduction

Tooth loss is a common clinical issue with functional, esthetic, and psychological impacts that can significantly affect patients’ quality of life (1–3). Rehabilitation of edentulism is still a clinical challenge, with treatment options including orthodontic treatments, removable or fixed prostheses, and dental implants. The choice among these therapeutic approaches depends on several factors, including patient age, skeletal maturity, alveolar bone dimensions and density, periodontal stability, the presence of healthy adjacent teeth, as well as overall treatment time and patient-centered considerations. Orthodontic space closure is most appropriate for growing patients because it can guide craniofacial development, although prolonged treatment duration is often poorly tolerated by adults (4). Implant-supported rehabilitation is contraindicated in young patients, as dental implants do not adapt to craniofacial growth and may result in long-term functional and aesthetic discrepancies (5–7). Furthermore, conventional prosthetic therapy can compromise pulpal vitality requiring endodontic treatment (8). In this context, tooth autotransplantation represents a biologically favorable and conservative alternative approach. Dental autotransplantation is defined as the transfer of a tooth from a donor site to a recipient site within the same individual. Indications include tooth loss resulting from complicated fractures, extensive carious lesions, or failed endodontic treatments, as well as congenital agenesis, impacted or ectopic teeth, post-extraction edentulous spaces, and trauma-related defects (8, 9). The main advantages of dental autotransplantation include preservation of proprioception through maintenance of the periodontal ligament, stimulation of alveolar bone remodeling during growth, the possibility of orthodontic alignment after transplantation, optimal biological compatibility, and lower treatment costs compared with prosthetic or implant-based rehabilitation (10–15). Successful outcomes depend on preserving periodontal ligament integrity, minimizing extra-alveolar time, performing atraumatic extraction, and ensuring adequate postoperative stabilization (16), together with careful case selection and meticulous radiographic and clinical planning. Despite its advantages, dental autotransplantation remains not widely adopt due to limitations in predicting donor tooth (DT) positioning, the requirement for advanced surgical expertise, and challenges in donor tooth selection. Recent improvements in digital dentistry - such as CBCT imaging, virtual planning, 3D modelling, and customized surgical guides - offer promising opportunities to enhance precision, reduce operative variability, and improve reproducibility (17, 18). However, evidence on the accuracy and clinical reliability of full-digital workflows for dental autotransplantation are still limited.

The purpose of this study was to assess the surgical accuracy of a full-digital workflow for dental autotransplantation. The primary aim was to evaluate the agreement between planned and achieved tooth positions at 18 months, clinical parameters were assessed to analyze the biological success of the procedure.

## 2 Materials and methods

### 2.1 Study design

The study was designed as a prospective cohort study. All procedures were conducted in accordance with the Declaration of Helsinki and were approved by the Regional Ethical Review Board of Central Calabria (Magna Graecia University of Catanzaro, Protocol No. 335/2024).

### 2.2 Study sample

Patients who simultaneously presented a non-restorable tooth (NRT) and at least one healthy, erupted third molar suitable as a donor tooth were enrolled in the study. All participants were classified as ASA I and were aged between 18 and 32 years, with fully developed roots of the third molars. Patients meeting any of the following conditions were excluded: allergy to corticosteroids or amoxicillin, immunosuppression, intravenous bisphosphonate therapy, psychiatric disorders, thrombocytopenia (<40,000/µL), coagulation disorders, severe hepatic cirrhosis, severe chronic renal failure, uncontrolled diabetes, and pregnancy or breastfeeding. Written informed consent was obtained from all participants prior to enrolment.

### 2.3 Preoperative preparation

At baseline, each patient underwent a comprehensive medical and dental history review followed by a clinical examination. Preoperative imaging included Cone-Beam Computed Tomography (CBCT; X-Mind Trium, Acteon® Group, Mérignac, France) with a field of view of 11 × 8 cm^2^, acquired at 85 kV and 8 mA (DAP: 782.554 mGy·cm^2^), and an intraoral scan (TRIOS Core, 3Shape® A/S, Copenhagen, Denmark) of the dental arches. Data were exported as Neuroimaging Informatics Technology Initiative (NIfTI) format for planning purposes.

### 2.4 Virtual surgical planning

**Figure 1:**
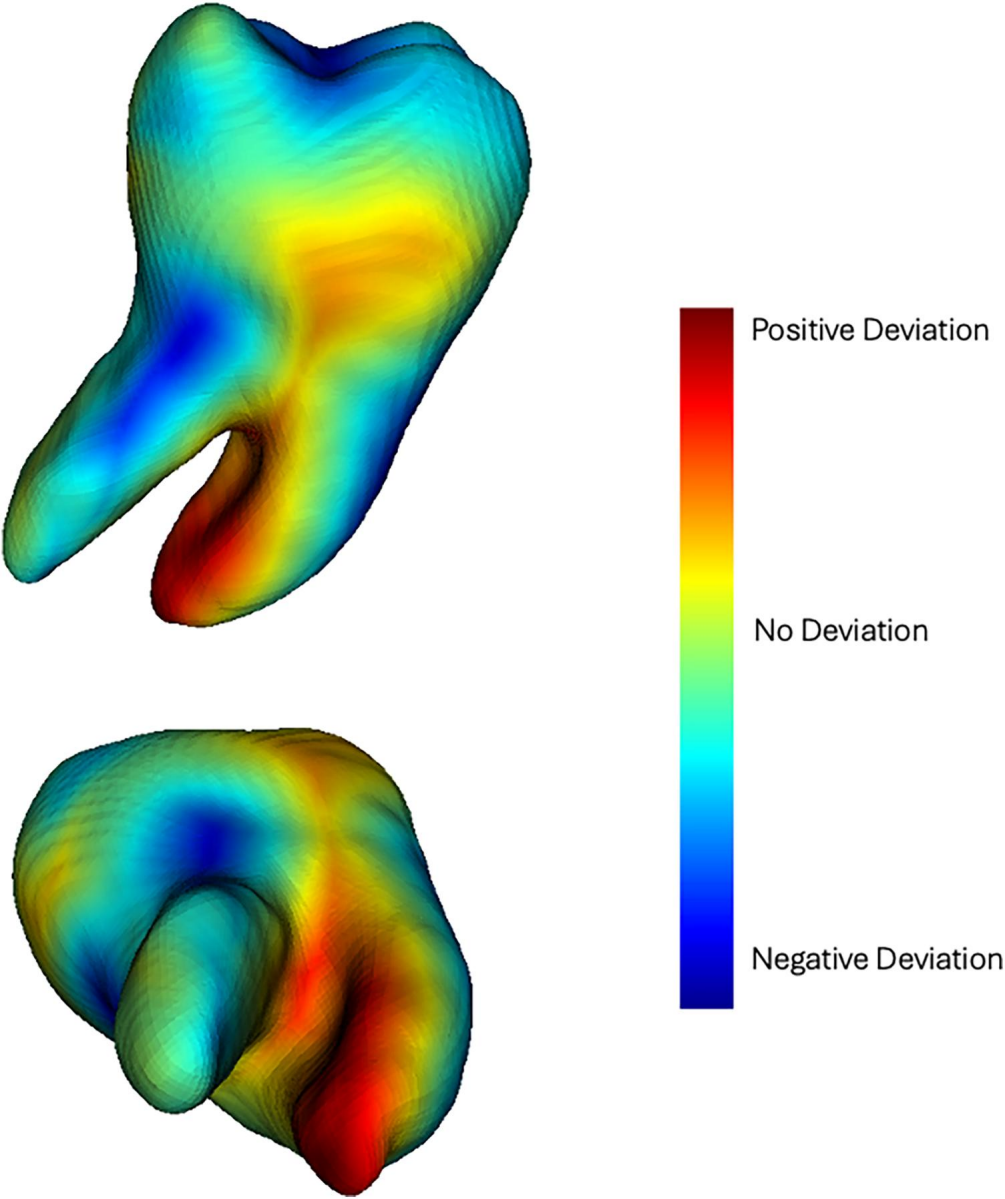
Colorimetric maps highlighting anatomical discrepancies between DT and NRT. Areas where DT is excessive compared to NRT are shown in red, while areas where it is in deficit are shown in blue.

**Figure 2:**
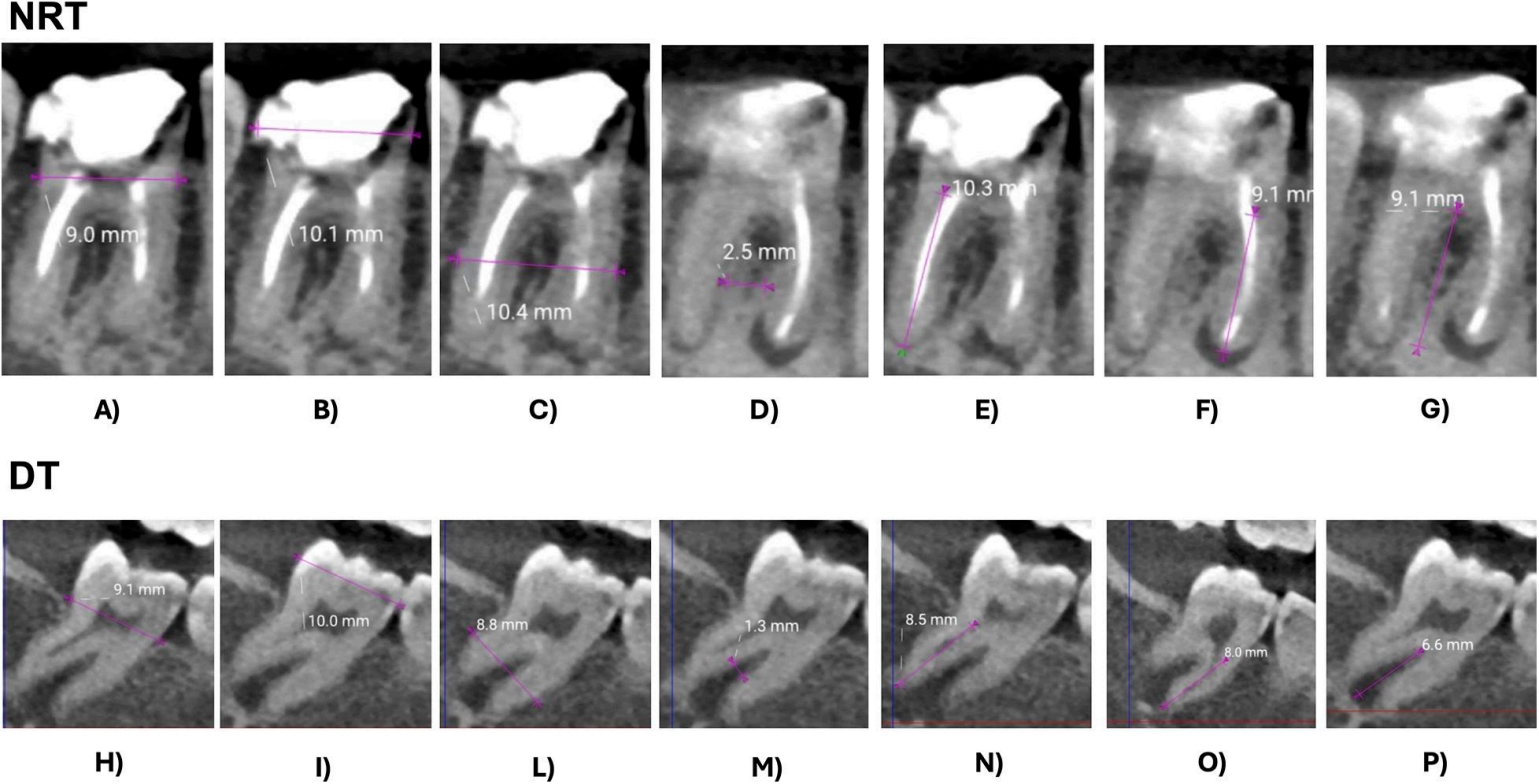
Measurements performed on NRT and DT: **(A–H)** mesio-distal CEJ width; **(B–I)** maximum mesio-distal crown width; **(C–L)** maximum mesio-distal root width; **(D–M)** maximum inter-radicular septum width; **(E–N)** mesial root length; **(F–O)** distal root length; **(G–P)** inter-radicular septum height.

The software 3D Slicer was used for digital identification and assessment of the donor tooth. Preoperative CBCT and intraoral scans were imported and oriented according to a standardized protocol based on the occlusal and midsagittal reference planes (19). Automatic segmentation of bones and teeth was performed using the DentalSegmentator extension, generating 3D stereolithographic (STL) models of the DT, NRT, and alveolar bone (20). Comparative analysis between the DT and the NRT was conducted using colorimetric maps to visualize morphological discrepancies: green represents similar morphology, red highlights regions where the DT exceeds the NRT, and blue indicates regions of deficit (Figure 1). Crown width, cervical dimensions, root length, and transverse root measurements were recorded from axial, sagittal, and coronal planes (Figure 2).

**Figure 3:**
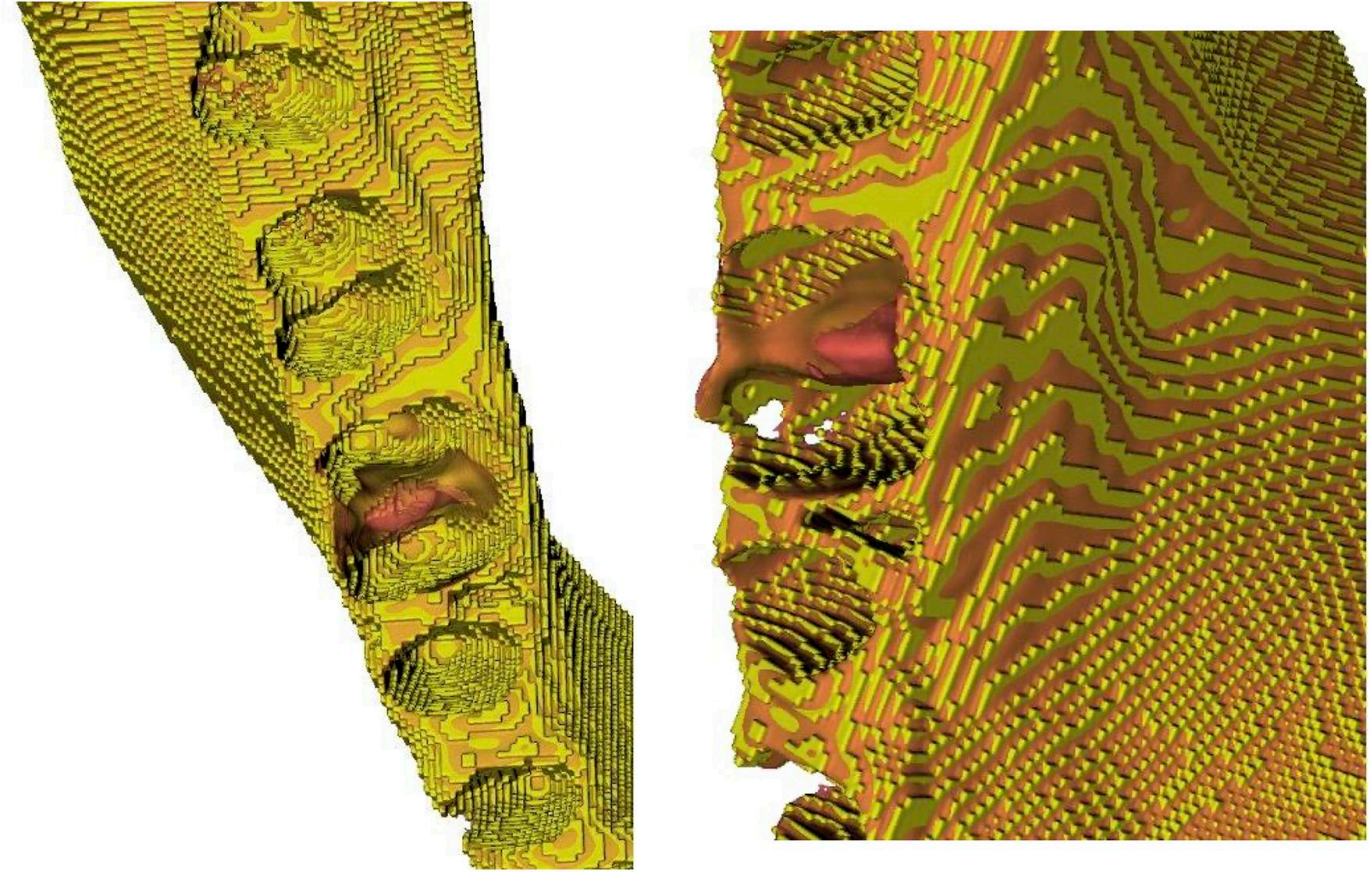
Morphological discrepancies of the unprepared alveolus (red) and virtually prepared alveolus (yellow).

Virtual autotransplantation was planned using RealGuide® software (3Diemme S.r.l., Cantù, Italy). Segmented models were imported to define the ideal angulation, rotation, and depth of the DT within the recipient site. A virtual subtraction analysis of the alveolar bone was carried out to simulate recipient site preparation based on the donor tooth morphology (Figure 3). The planned tooth positions led to the design of customized surgical guides, which accurately replicated the digitally planned ostectomy path. DT replicas and surgical guides were fabricated using a FormLabs 3B printer (Formlabs®, Somerville, MA, USA) with its proprietary Surgical Guide resin (FormLabs®, Somerville, MA, USA), a biocompatible and sterilizable material with high dimensional stability (21, 22).

### 2.5 Clinical procedure

One week before surgery, all patients underwent professional oral hygiene to reduce bacterial contamination, and interproximal separators were placed adjacent to both the NRT and the DT to facilitate the dental extraction. Prophylactic therapy was administered 1 h before surgery (2 g amoxicillin/clavulanic acid or 600 mg clindamycin for penicillin-allergic patients). Immediately before the procedure, patients rinsed with 0.2% chlorhexidine gluconate solution (Curasept® ADS, Curaden Healthcare, Switzerland) for one minute.

All procedures were performed under local anaesthesia (mepivacaine 20 mg/mL with epinephrine 1:100,000 - Pierrel, Milan, Italy) by the same experienced operator (A.G). The NRT was extracted performing a flapless, atraumatic technique with controlled luxation to preserve the alveolar bone. Surgical guides were placed to perform the alveolar bone preparation according to the virtual plan. The DT replica was used to check that the alveolar osteotomy matched the DT morphology.

**Figure 4:**
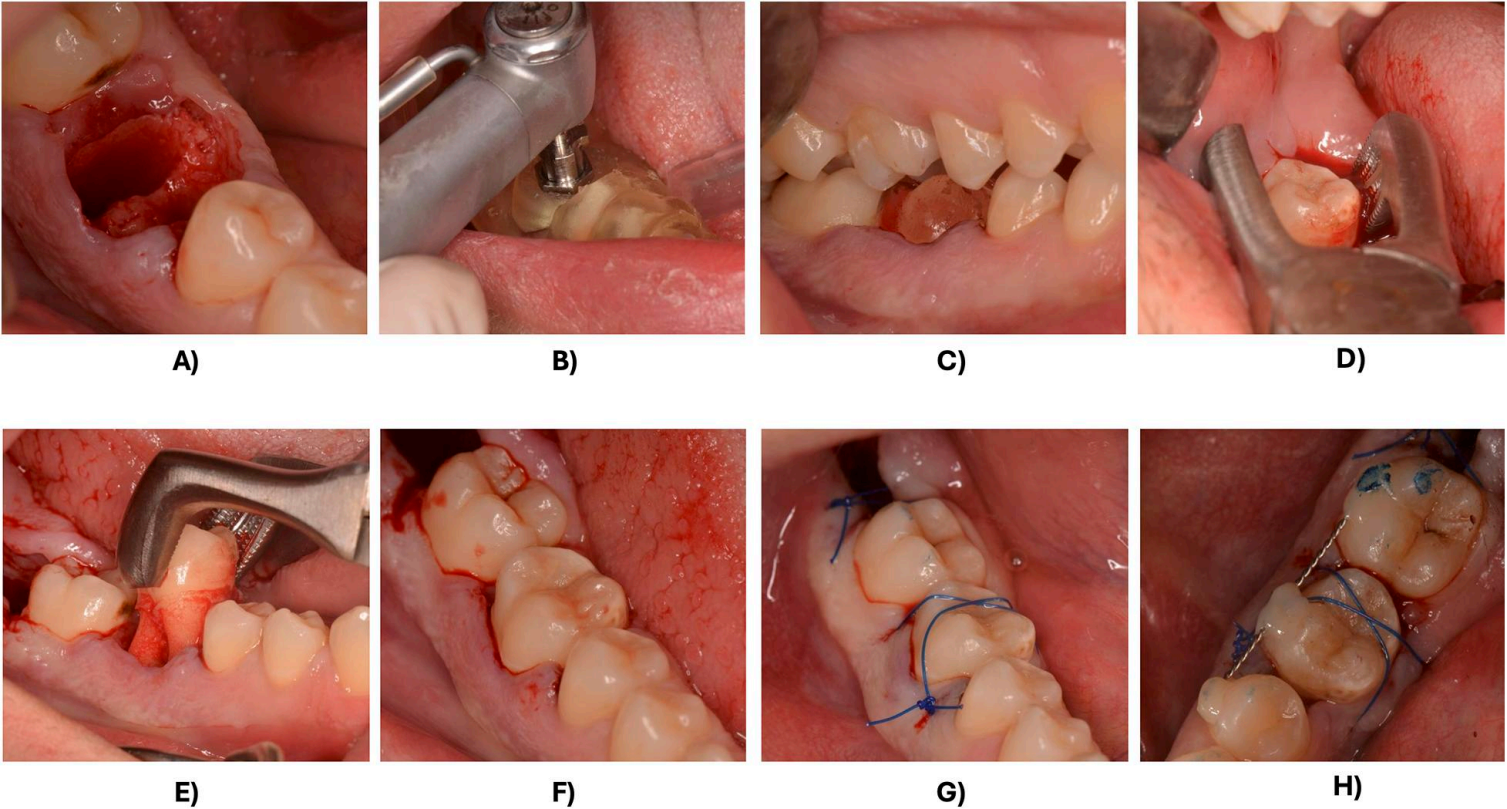
Surgical procedure: **(A)** minimally invasive extraction of the NRT; **(B)** osteotomy performed using guided surgery; **(C)** placement of the 3D-printed prototype of the DT; **(D)** atraumatic extraction of the DT; **(E,F)** transplantation of the DT; **(G)** suture; **(H)** splinting.

The DT was atraumatically extracted using forceps, while elevators were used only minimally during the initial stages to preserve the periodontal ligament (PDL) integrity. The DT was carefully placed in the prepared socket in slight infraocclusion. Extra-alveolar time was minimized and recorded in all cases. Stabilization was achieved using nonabsorbable polypropylene sutures (Surgipro®, Braun, Melsungen, Germany) and an orthodontic splint bonded passively to the buccal and lingual surfaces. Periapical radiographs were immediately performed after splinting using a paralleling technique with a beam-aiming device to confirm proper seating of the DT. The transplanted tooth remained splinted for 3 weeks (Figure 4).

### 2.6 Post-surgical management and follow-up

All patients received the same antibiotic therapy for 6 days (amoxicillin/clavulanic acid 875/125 mg or clindamycin 600 mg) and rinsed with 0.2% chlorhexidine twice daily for 1 week. Analgesics (paracetamol 1 g as needed) were prescribed to manage postoperative discomfort, and patients were advised to avoid chewing on the side of the transplanted tooth until sufficient healing had occurred. After surgery, a gel containing 1% chlorhexidine and hyaluronic acid (Curasept, Curaden Healthcare, Switzerland) was applied directly to the surgical site as instructed. Follow-up evaluations were scheduled at 1, 2, and 4 weeks, and at 3, 6, 12, and 18 months. Periapical radiographs were obtained at 1, 6, and 12 months, while a CBCT scan was performed at 18 months. Hygiene sessions have been scheduled every 4 months.

### 2.7 Data analysis workflow and study variables

Eighteen-month after surgery, a new CBCT was acquired to evaluate both the health status of the transplanted tooth and the accuracy of the surgical planning. Postoperative NIfTI files were imported into 3D Slicer and aligned on the preoperative data using the Landmark Registration tool. Automatic segmentation generated postoperative 3D models of the osteotomized alveolar bone and of the DT. Comparative analysis between planned and postoperative models was conducted using the ModelToModelDistance extension. Deviations were visualized as colorimetric maps and qualitatively assessed on ShapePopulationViewer. Quantitative analysis included mean linear surface deviations, as well as linear and angular deviations along the X, Y, and Z axes, automatically calculated using the AQ3DC plugin. Landmarks were identified based on tooth morphology, with two coronal and two apical landmarks for multirooted teeth and one coronal and one apical landmark for single-rooted teeth. The primary outcome was the spatial deviation between planned and post-surgical models. Secondary outcomes included surgical time, extra-alveolar times, and clinical parameters, such as mobility, percussion sensitivity, pulp vitality, probing depth, tooth colour, and masticatory function, systematically evaluated at each follow-up visit.

### 2.8 Statistical analysis

Data were recorded in a dedicated Excel (Microsoft, Redmond, WA, USA) file and analysed using RStudio (RStudio, PBC, Boston, MA, USA). Categorical variables were described using absolute and percentage distributions, while continuous variables were reported as means ± standard deviations. Linear regression analysis was performed to assess the correlation between coronal and apical displacements, as well as between linear and angular displacements. The significance level was set at *α* < 0.05.

## 3 Results

**Figure 5:**
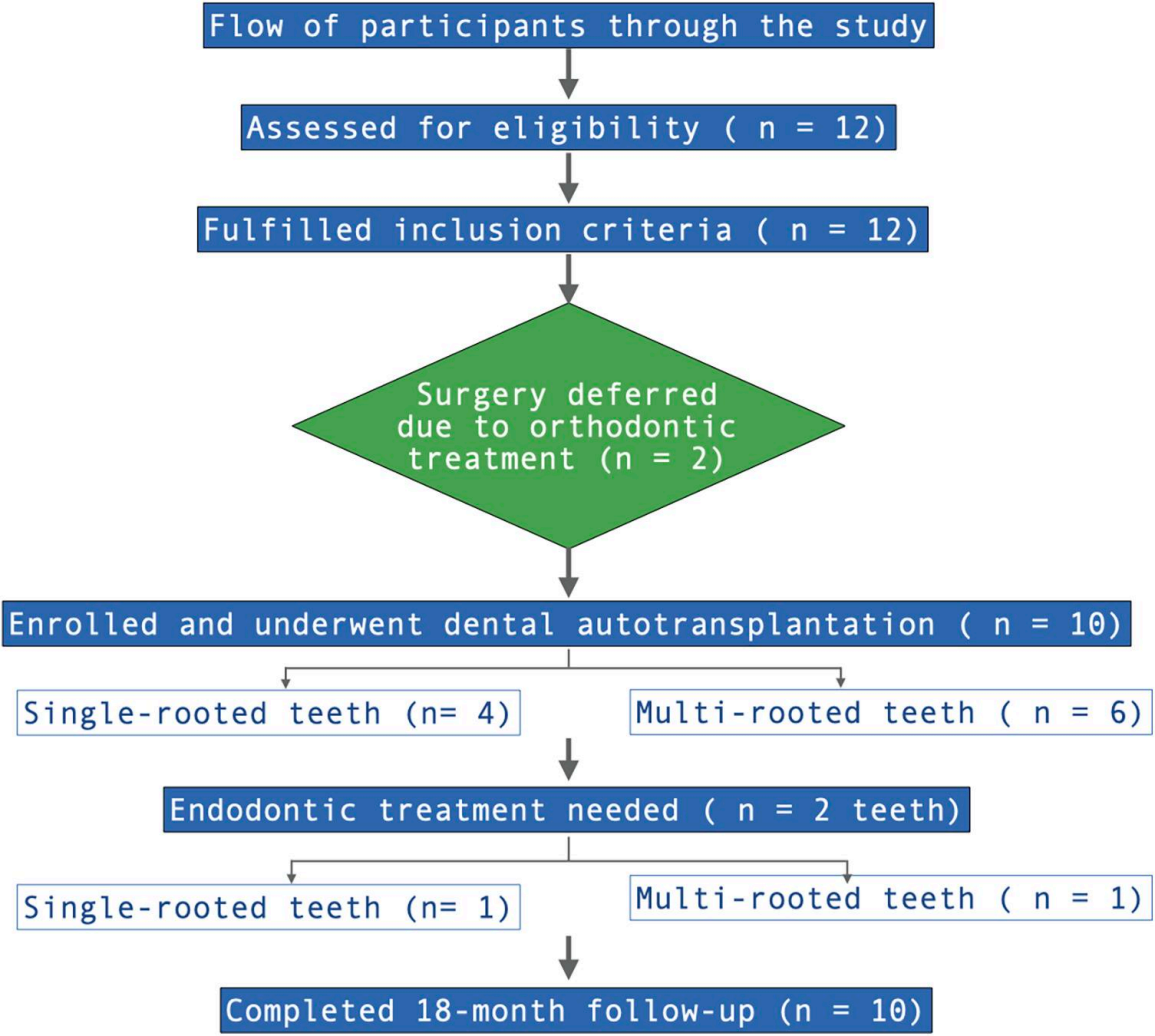
Study flowchart showing patient enrollment and follow-up.

During the clinical study period (May 2024 – December 2025), 12 patients attending the Oral Surgery Clinic of the Department of Dentistry, Magna Graecia University of Catanzaro, were initially enrolled. All patients fulfilled the inclusion criteria defined in the study protocol; in two cases, surgery was deferred to allow completion of a preliminary orthodontic treatment recommended by the therapeutic plan (Figure 5). Consequently, the final sample included 10 patients (6 males, 4 females) with a mean age of 27.2 ± 2.77 years. Donor teeth included both single- and multi-rooted types: four cases involved tooth 1.8 (single-rooted), four cases tooth 4.8 (multi-rooted), and two cases tooth 2.8 (multi-rooted). All donor teeth exhibited complete root development. The mean extra-alveolar time was 7 ± 19 s, and the mean surgical time was 26.7 ± 12.1 min (Table 1). Endodontic treatment was performed only when clinically indicated. In two cases, donor teeth required post-transplant endodontic treatment due to the presence of periapical lesions, involving one multirooted and one single-rooted teeth. Eighteen-month follow-up highlighted good clinical outcomes, with stable transplanted teeth, physiological probing depths, absence of pathological mobility, and no radiographic signs of resorption or periapical alterations. All non-endodontically treated teeth showed positive responses to vitality testing, suggesting generally preserved pulp sensibility.

**Table 1:** Descriptive statistics of the study sample.

Variables	Study sample
Patients included	10
Sex	
Males (%)	6 (60%)
Females (%)	4 (40%)
Mean age (years)	27,2 ± 2,77
Donor teeth	
1.8 (single-rooted)	4 (40%)
4.8 (multi-rooted)	4 (40%)
2.8 (multi-rooted)	2 (20%)
Endodontic therapy	
1.8 (single-rooted)	1
4.8 (multi-rooted)	1
2.8 (multi-rooted)	0
Mean extraoral time (s)	7 ± 19
Mean surgical time (min)	26,7 ± 12,1

### 3.1 Qualitative and quantitative analysis

**Figure 6:**
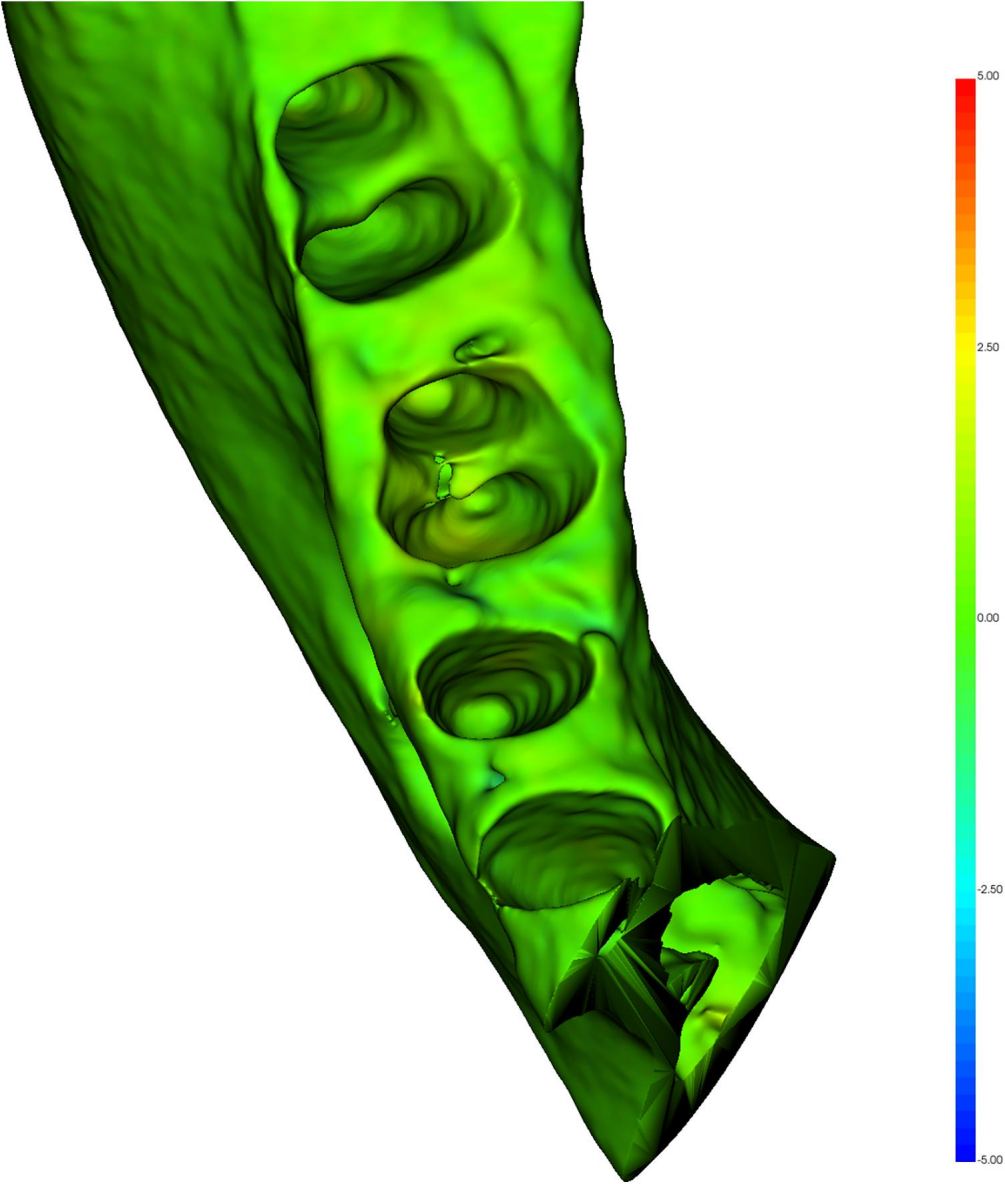
Qualitative analysis of alveolar bone. Areas that deviate from the virtual preparation of the alveolus are shown in yellow.

**Figure 7:**
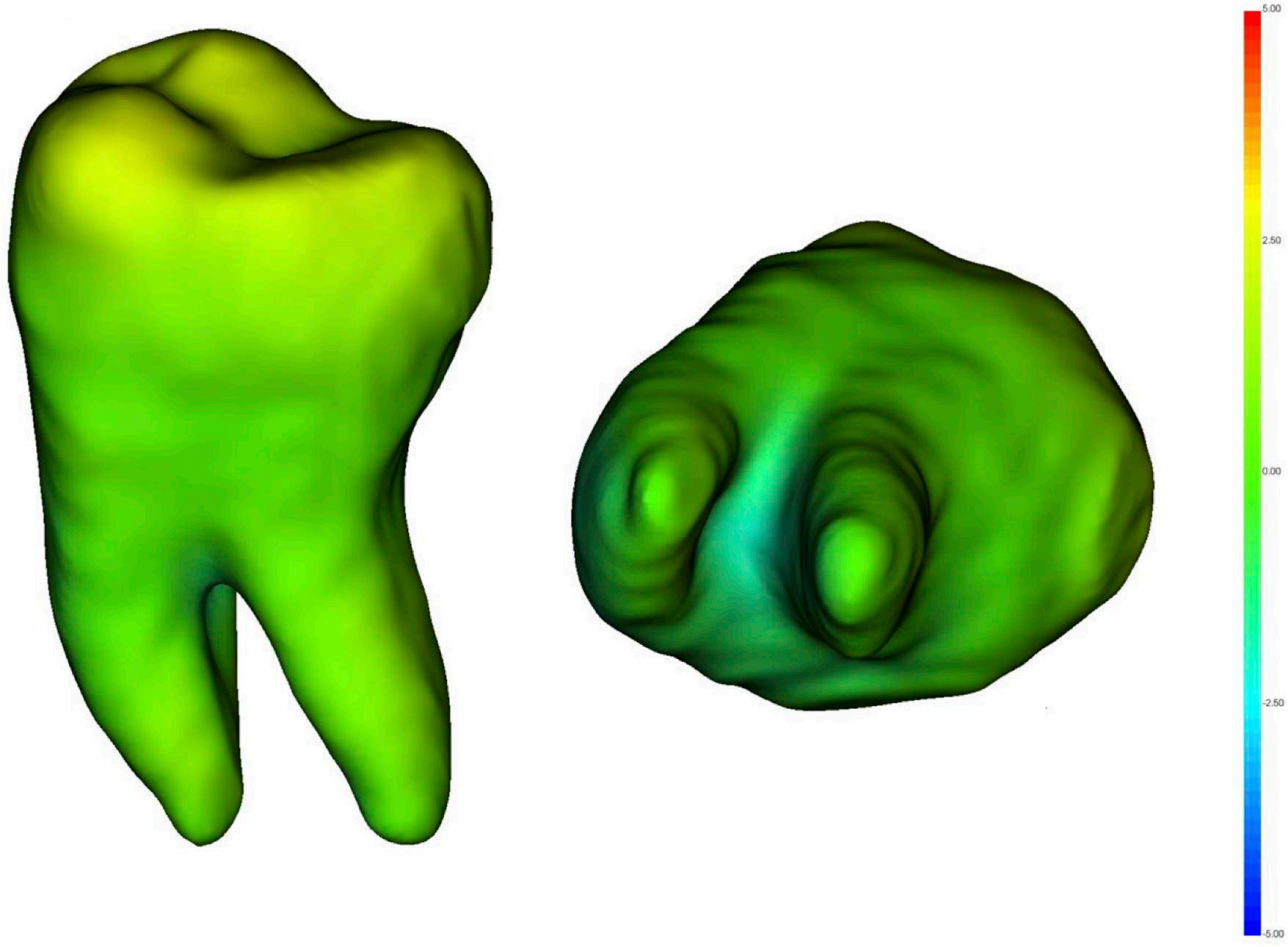
Qualitative analysis of DT. The areas that differ from the digital plan appear in blue.

**Figure 8:**
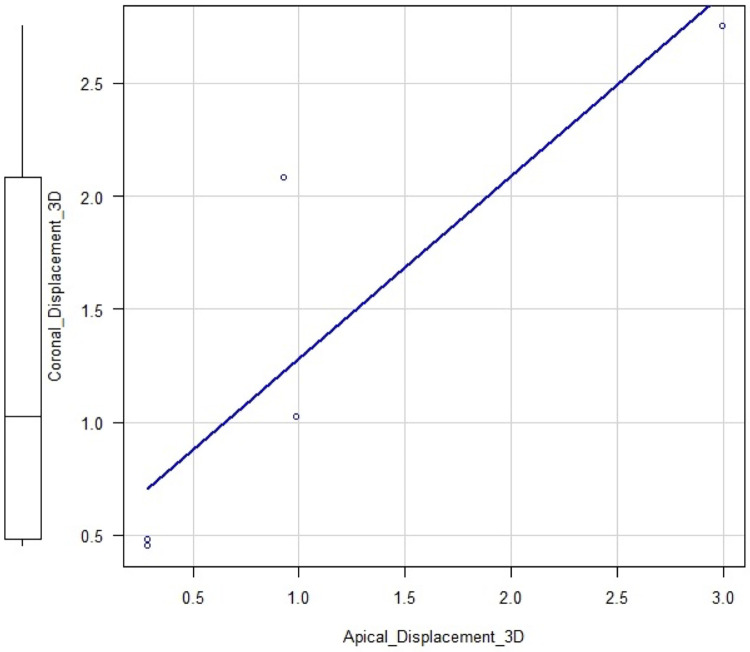
Linear regression model showing positive correlation between coronal and apical displacement of the DT (regression slope = 0.96 ± 0.29).

The qualitative assessment revealed minor deviations between the planned and post-surgical outcomes. For bone assessment, the colormaps showed a slight morphological discrepancy (yellow areas) mainly at the level of the interradicular septum (Figure 6). Qualitative analysis of the DT revealed greater surface heterogeneity in the colorimetric maps than in the bone analysis, with variations observed mainly in the interradicular region (Figure 7). Quantitative analysis revealed a mean surface deviation of 0.63 ± 0.45 mm (tooth) and 0.17 ± 0.06 mm (alveolar bone). Linear deviations at coronal and apical levels along X, Y, and Z axes were all <2 mm, with overall displacements of 2,04 ± 1,24 mm (coronal) and 1,36 ± 1,00 mm (apical) (Table 2). Higher angular deviations (yaw, pitch, roll) were found for Yaw (Table 2). Linear regression showed a positive correlation between coronal and apical displacements (intercept = −0.21; t = 3.23; *p* = 0.048; adjusted R-squared: 0.70), indicating consistent axial movement (Figure 8). No significant correlation was found between linear and angular displacements (*p* > 0.05).

**Table 2:** Angular and linear displacements: mean ± standard deviation and [95% CI].

Angular measurements [°]				
	Yaw	Pich	Roll	-
Discrepancy	−6,06 ± 10,84 [−19,5;7,39]	1,86 ± 2,35 [−1,06;4,77]	−2,68 ± 5,8 [−9,87;4,51]	-
Linear measurements [mm]				
	*X* axis	*Y* axis	Z axis	3D displacement
Coronal displacement	0,06 ± 2,07 [−2,51;2,63]	0,59 ± 1,20 [−0,89;2,07]	−0,06 ± 0,64 [−0,86;0,74]	2,04 ± 1,24 [0,49;3,58]
Apical displacement	−0,47 ± 0,96 [−1,65;0,72]	0,63 ± 1,00 [−0,61;1,87]	0,39 ± 0,60 [−0,35;1,13]	1,36 ± 1,00 [0,12;2,61]
Automated surface displacements [mm]				
Tooth surface displacement	0,60 ± 0,45 [−0,01;1,21]			
Mandible surface displacement	0,17 ± 0,06 [0,09;0,25]			

## 4 Discussion

This study aimed to evaluate the accuracy of a new procedure for dental autotransplantation using a 3D full-digital workflow. The primary outcome was the spatial deviation between planned and post-surgical models of the DT and the receipt alveolar bone. Both qualitative and quantitative assessments were performed through comparative analysis, with specific focus on DT selection and the predictability of its long-term clinical integration.

Colorimetric map analysis showed high degree of correspondence between the planned and post-surgical models, with discrepancies observed at the interradicular septum. Surface deviations were minimal for both the tooth and the alveolar bone. Linear discrepancies across the three spatial axes were overall less than 2 mm, while rotational deviations remained under 3°, except for rotations occurring in the axial plane. The observed coronal deviation is within a biologically acceptable range, as minor discrepancies between planned and actual tooth position do not compromise PDL vitality or short- to mid-term stability when atraumatic insertion and minimal extra-alveolar time are ensured (23). The observed axial rotation likely represents a controlled intraoperative adjustment, facilitating passive three-dimensional adaptation of the DT to the alveolar bone and avoiding the need for rigid positioning guides. These results align with findings by Zhang et al., who reported clinical success of autotransplantation at 12 months of follow-up with linear and angular deviations within 2 mm and 7°, respectively (24). Rigid templates were previously proposed by Shahbazian et al. using a prototype 3D-printed tooth with occlusal support guides (25, 26). These devices can accurately transfer the digitally planned tooth position; however, their rigid design may constrain the root, generating excessive stress on the alveolar walls and increased mechanical loading on the PDL, which can compromise biological healing and predispose tooth to root resorption (14, 15, 27–29). In the present workflow, a 3D replica of the DT was used without rigid templates, allowing controlled micro-movements and improving three-dimensional integration.

A fundamental preoperative step of this digital workflow was the automated morphological assessment of the DT and NRT, enabling a reliable evaluation of anatomical compatibility and identification of areas of correspondence and divergence between the two models (24, 30). Measurements on the axial, sagittal, and coronal planes quantified millimetric discrepancies between DT and NRT, with particular attention to the enamel-cement junction and the interradicular septal bone. These anatomical structures are recognized as key predictors of donor-recipient compatibility, critical for achieving primary stability of the tooth (31, 32). Nevertheless, over-preservation of the interradicular septum or mismatched alveolar dimensions have been associated with increased incidence of ankylosis and delayed periodontal healing in multiple clinical series (33, 34).

A relevant feature of the study was the possibility of simulating the surgical preparation of the recipient site preoperatively, precisely defining the bone volume to be removed. To the best of our knowledge, no previously published studies have described approaches that integrate three-dimensional previewing of alveolar site preparation within the context of dental autotransplantation. This allowed for precise preparation of the recipient site, avoiding both under-preparation, which could have interfered with passive positioning, and over-preparation, which could have compromised primary stability and periodontal vitality, facilitating a biologically advantageous adaptation between the donor tooth and the alveolar walls. Such controlled adaptation of root and bone dimensions has been consistently recognized in the literature as essential for promoting favorable transplant outcomes, whereas inappropriate socket dimensions are associated with higher complication rates (15, 18, 25, 35–37). Specifically, quantitative studies suggest that maintaining a marginal distance of 0.2–0.5 mm between the donor root and the alveolar bone reduces mechanical stress on the PDL and decreases early resorption rates (23).

This workflow also enabled the development of patient-specific, root-oriented surgical guides (14, 37–39). Although Yau et al. previously demonstrated the effectiveness of customized surgical guides in dental autotransplantation, the guided surgery protocol adopted in their study did not include a three-dimensional preview of the alveolar site preparation (14). Notably, Lucas-Taulé et al., in a prospective study, recorded the efficiency of multi-angle guides for selective preparation of the recipient site, reporting clinical stability and no complications in the long term (40). Nevertheless, the adoption of multi-angle surgical guides with defined drilling trajectories, although providing high precision in alveolar site preparation, may reduce intraoperative flexibility and increase procedural complexity, potentially compromising the achievement of an optimal anatomical congruence between donor and recipient sites (18, 34). Our protocol was based on detailed digital simulation of bone removal to generate an alveolar geometry and volume specifically tailored to the root morphology of the DT, thereby minimizing the number of surgical templates required and enabling faster, less invasive procedures. These observations are in line with evidence that customized guides and digital planning reduce extra-alveolar time and root trauma, improving long-term survival and reducing the incidence of post-transplant endodontic treatment (18, 23). The clinical results obtained at 18-months follow-up support the notion that full-digital planning not only enhances surgical precision but was also associated with favorable mid-term biological outcomes under controlled clinical conditions (33, 41, 42).

The clinical success was confirmed by the physiological probing depths, the absence of pathological mobility, and no radiographic signs of resorption or periapical alterations. Surgical precision aimed at minimizing extra-alveolar time is a cornerstone of the protocol, as preservation of the periodontal ligament is crucial for periodontal healing and long-term biological success.

Limitations of the study include the small sample size, due to the highly specialized nature of dental autotransplantation and selective inclusion criteria, as well as the short-term follow-up, which limited the assessment of long-term survival and late complications. Although 18 months does not reflect long-term survival, it is adequate to identify early biological complications, such as inflammatory root resorption, pulp necrosis, or initial signs of ankylosis. Longer follow-up is required to evaluate late replacement resorption and long-term stability (23, 42, 43).

Future studies should expand the cohort and extend follow-up to validate reproducibility and long-term outcomes of this protocol. Furthermore, the adoption of this workflow is strictly linked to the availability of advanced digital technologies and three-dimensional printing operator experience, potentially limiting its broader generalizability. Nevertheless, the longer planning time is compensated by reduced intraoperative duration and lower procedural invasiveness. Continued development of AI-driven segmentation and imaging technologies is expected to further improve workflow reliability and accessibility, supporting broader adoption of digital-assisted, minimally invasive dental autotransplantation protocols.

## 5 Conclusion

This preliminary study suggests the clinical feasibility of a fully digital workflow for dental autotransplantation. Within the limits of the small sample size, the findings indicate a close correspondence between virtual planning and surgical execution, with limited spatial deviations and favorable mid-term outcomes.

The exclusive use of digital planning was associated with reduced operator-dependent variability and enhanced procedural standardization. The close correspondence observed between planned and final tooth positions appears promising for the potential of fully digital workflows as a predictable and biologically oriented approach to dental autotransplantation.

## Data Availability

The datasets generated for this study are not publicly available due to ethical and privacy restrictions related to the use of patient data. Requests to access the datasets cannot be accommodated. Requests to access the datasets should be directed to selene.barone@unicz.it.
